# CDC’s Emergency Management Program Activities — Worldwide, 2003–2012

**Published:** 2013-09-06

In 2003, recognizing the increasing frequency and complexity of disease outbreaks and disasters and a greater risk for terrorism, CDC established the Emergency Operations Center (EOC), bringing together CDC staff members who respond to public health emergencies to enhance communication and coordination. To complement the physical EOC environment, CDC implemented the Incident Management System (IMS) (1,2), a staffing structure and set of standard operational protocols and services to support and monitor CDC program-led responses to complex public health emergencies. The EOC and IMS are key components of CDC’s Emergency Management Program (EMP) (3), which applies emergency management principles to public health practice. To enumerate activities conducted by the EMP during 2003–2012, CDC analyzed data from daily reports and activity logs. The results of this analysis determined that, during 2003–2012, the EMP fully activated the EOC and IMS on 55 occasions to support responses to infectious disease outbreaks, natural disasters, national security events (e.g., conventions, presidential addresses, and international summits), mass gatherings (e.g., large sports and social events), and man-made disasters. On 109 other occasions, the EMP was used to support emergency responses that did not require full EOC activation, and the EMP also conducted 30 exercises and drills. This report provides an overview of those 194 EMP activities.

What is already known on this topic?

Since 2003, CDC’s Emergency Management Program (EMP) has implemented standard incident management protocols and procedures and established an emergency operations center (EOC) to support field investigation, information interchange, and logistics functions required to effectively respond to complex public health emergencies.

What is added by this report?

During 2003–2012, the EOC had 55 activations and 109 utilizations. The EMP responded to a wide range of domestic and international emergencies, including infectious disease outbreaks and natural and man-made disasters.

What are the implications for public health practice?

Public health agencies’ use of standardized, centralized, and structured systems and protocols, such as EOCs and Incident Management Systems, can improve emergency response capability and provide increased health security.

The EMP can access all of CDC’s organizational resources, enabling synchronization of public health emergency response activities and communications with international, federal, and state partners. EMP public health response activities are categorized as activations, utilizations, exercises and drills (Figure 1), and public health triage. Activations must be approved by the CDC Director and include use of the IMS, which includes the gathering of key staff members from across CDC to the EOC, coordination of planning and communications, logistics support, and field deployments (e.g., to a hurricane-damaged country) for a comprehensive agency-wide response. Utilization does not always require full EOC activation, but employs EMP services to meet the needs of the situation, such as call center operations, development of plans and situational awareness products, and travel assistance. Exercises and drills include full-scale emergency response exercises (deployment of staff and materiel to support a scenario that mimics a real emergency), tabletop exercises (discussion of a scenario), and drills (tests of a single response function). At all times, public health triage is used with telephone call and e-mail requests, linking CDC subject matter experts and resources with key partners, such as state and local health departments, other federal agencies, and public health practitioners.

During activations and exercises, IMS staff structures and protocols are used to support a standardized but flexible approach to CDC’s public health response. Use of the IMS response model allows CDC to stay consistent with the incident command structure used by other agencies (4). The CDC center, institute, or office with primary responsibility for the public health problem being addressed (e.g., infectious disease or natural disaster) leads the response using the support structure and resources coordinated by the EMP.

During 2003–2012, CDC supported 55 activations, with an average activation period of 52.9 days (1–394 days [excluding the ongoing polio activation]). The most common type of activation was for infectious disease outbreaks (22, 40.0%), including seven (31.8%) associated with respiratory illness. Natural disasters accounted for 16 (29.0%) of the activations, most commonly hurricanes (11, 68.8%). In addition, the EOC and IMS were activated to support the response to nine man-made disasters, seven national security events, and one mass gathering (Table). The longest activation to date has been the ongoing support of the international polio eradication campaign (643 days as of September 6, 2013).

Forty-one (74.6%) of the 55 activations were for responses occurring within the United States (Table). During this period, the activations for infectious disease outbreak responses most often were initiated in December; natural disaster activations occurred most commonly in August (Figure 2). Support functions were used 109 times for public health events not requiring activation (Figure 1). These included support for infectious disease outbreak investigations (52 times, 47.7%), natural disasters (31, 28.4%), monitoring of national security events (17, 15.6%) and mass gatherings (nine, 8.3%). The most common utilization events were for foodborne disease outbreaks (25). Among the 109 utilizations, 72 (66.1%) occurred in the United States (Table). The average duration of all utilizations was 10 days (range: 1–92 days).

The EMP either coordinated or was an integral participant in 30 full-scale or tabletop exercises and drills during this 10-year period. Twelve (40%) of the exercises and drills used terrorism event scenarios to test public health system response capabilities. In addition to these 30 exercises and drills, the EMP provided support for many exercises and drills conducted by its federal, state, and local emergency response partners.

Another important service offered by the EMP is its availability 24 hours a day, 365 days of the year to international and domestic partners for referral of telephone and e-mail requests for technical assistance and public health consultation. During 2004–2012, EOC watch officers triaged an average of 23,303 requests per year (range: 14,633–38,812).

The EMP provides technical assistance to multiple countries interested in learning more about EOC operations and use of the IMS. In 2013, the EMP sponsored its first five international emergency management fellows. The EMP also supports the Global Health Security Demonstration Project, an initiative conducted in partnership with the governments of Vietnam and Uganda and the World Health Organization, to build capacity for surveillance and detection and response to epidemics. The project has focused on developing plans to build additional EOCs and emergency response capability and to provide laboratory and information technology infrastructure to support global health security.

## Reported by

*Laura Leidel, MSN, MPH, Samuel L. Groseclose, DVM, Office of Science and Public Health Practice; Bruce Burney, MEd, Phil Navin, MA, Mark Wooster, PhD, Div of Emergency Operations, Office of Public Health Preparedness and Response, CDC.* ***Corresponding contributor:*** *Laura Leidel, lleidel@cdc.gov, 770-488-5991.*

## Editorial Note

Preparing for and protecting against public health threats is a key aspect of CDC’s mission, both domestically and around the world. The EMP has been used regularly for response to public health threats, including full activation of the EOC and use of the IMS structure or use of selected EMP support services, over the past 10 years. Simultaneously, the EMP has increased the number of drills and exercises that it supports to aid CDC programs in planning and preparedness activities. In recent years, the protocols and services provided by the EMP have been modified in response to findings from after-action evaluations and surveys (Division of Emergency Operations, Office of Public Health Preparedness and Response, CDC, unpublished data, 2003–2012) to better meet the special needs of public health responses. Results from a 2011 EMP stakeholder survey (Division of Emergency Operations, Office of Public Health Preparedness and Response, CDC, unpublished data, 2011) indicated increasing awareness of the benefits of using the EMP for public health emergency response. Survey results also indicated the need for additional staff training in the use of the IMS for responses.

The uniqueness of emergency events and the multiple factors that influence their course make it challenging to measure the effectiveness of the EMP. Measures of performance and cost effectiveness associated with preparedness and response have not been clearly defined. In 2012, to assess and strengthen the emergency response, CDC began working toward agencywide accreditation by the Emergency Management Accreditation Program (EMAP). Participation in EMAP has allowed the EMP to begin to identify metrics to assess performance and the cost effectiveness of response activities.

Continued review of the EMP activations, utilizations, and exercises and drills will help CDC better understand and address the needs of its stakeholders, both domestic and international. Further evaluation of the effectiveness of the EMP and training in the use of the EOC and IMS protocols is needed for continued program improvements. Identifying and addressing the challenges faced by CDC staff members when engaged in EMP activities will improve CDC’s ability to respond effectively and further strengthen the nation’s health security.

## Figures and Tables

**FIGURE 1:**
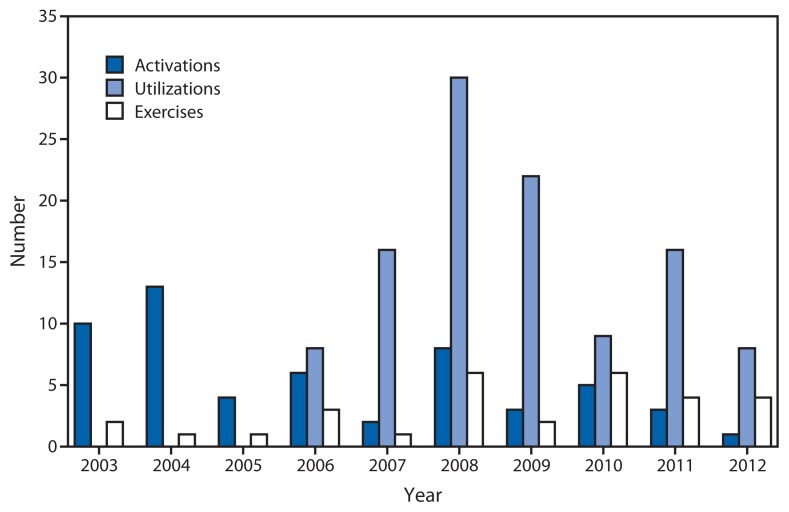
Number of activations, utilizations, and exercises, by year initiated — Emergency Management Program, CDC, 2003–2012

**FIGURE 2:**
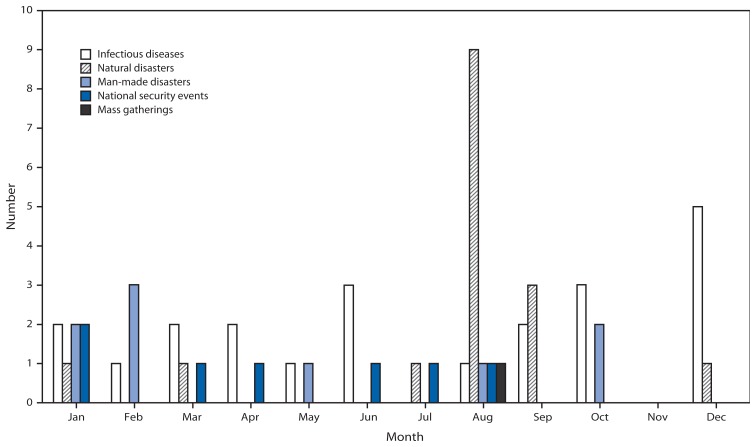
Number of activations (N = 55), by month initiated and cause — Emergency Management Program, CDC, 2003–2012

**TABLE:** Number of public health emergency activities (N = 194), by type, cause, and location — Emergency Management Program, CDC, 2003–2012

Activity type/Cause	Location			Total

	Within United States	Outside United States	Both	
**Activations (n = 55)**				
**Infectious disease (40.0%)**	**14**	**6**	**2**	**22**
Respiratory	3	2	2	**7**
Foodborne	7	0	0	**7**
Vaccine preventable	1	2	0	**3**
Viral hemorrhagic fever	0	1	0	**1**
Fungal	1	0	0	**1**
Vectorborne	1	0	0	**1**
Waterborne	0	1	0	**1**
Zoonotic	1	0	0	**1**
**Natural disaster (29.0%)**	**12**	**3**	**1**	**16**
Hurricane	10	0	1	**11**
Earthquake	0	3	0	**3**
Tropical storm	2	0	0	**2**
**Man-made disaster (16.4%)**	**8**	**0**	**1**	**9**
Bioterrorism	3	0	0	**3**
Space debris	1	0	1	**2**
Toxic spill	2	0	0	**2**
Blackout	1	0	0	**1**
Wildfire	1	0	0	**1**
**National security event (12.7%)**	**7**	**0**	**0**	**7**
**Mass gathering (1.8%)**	**0**	**1**	**0**	**1**
**Total**	**41**	**10**	**4**	**55**
**Utilizations (n = 109)**				
**Infectious disease (47.7%)**	**28**	**24**	**0**	**52**
Foodborne	18	7	0	**25**
Viral hemorrhagic fever	0	8	0	**8**
Waterborne	2	5	0	**7**
Respiratory	3	3	0	**6**
Vectorborne	2	1	0	**3**
Vaccine preventable	3	0	0	**3**
**Natural disaster (28.4%)**	**23**	**8**	**0**	**31**
Flood	6	3	0	**9**
Tropical storm	6	0	0	**6**
Earthquake	0	3	0	**3**
Hurricane	2	1	0	**3**
Volcano	2	1	0	**3**
Tornado	2	0	0	**2**
Ice storm	2	0	0	**2**
Unusual substance	2	0	0	**2**
Wildfire	1	0	0	**1**
**National security event (15.6%)**	**15**	**2**	**0**	**17**
**Mass gathering (8.3%)**	**6**	**3**	**0**	**9**
**Total**	**72**	**37**	**0**	**109**
**Exercises and drills (n = 30)**				
**Full-scale exercise (66.7%)**	**15**	**0**	**5**	**20**
Terrorism	7	0	0	**7**
Infectious disease	1	0	4	**5**
Multiple scenarios	4	0	0	**4**
Natural disaster	3	0	0	**3**
Man-made disaster	0	0	1	**1**
**Tabletop exercise (26.7%)**	**6**	**0**	**2**	**8**
Terrorism	4	0	1	**5**
Infectious disease	1	0	1	**2**
Natural disaster	1	0	0	**1**
**Drill, not specified (6.7%)**	**2**	**0**	**0**	**2**
**Total**	**23**	**0**	**7**	**30**
